# Evaluation of a Cognitive Rehabilitation Protocol in HIV Patients with Associated Neurocognitive Disorders: Efficacy and Stability Over Time

**DOI:** 10.3389/fnbeh.2015.00306

**Published:** 2015-11-16

**Authors:** Alessandro Livelli, Gian Carlo Orofino, Andrea Calcagno, Mariana Farenga, Donatella Penoncelli, Marta Guastavigna, Sinibaldo Carosella, Pietro Caramello, Lorenzo Pia

**Affiliations:** ^1^Division A of Infectious Diseases, Amedeo of Savoia HospitalTorino, Italy; ^2^SpAtial, Motor and Bodily Awareness Research Group, Department of Psychology, University of TorinoTorino, Italy; ^3^Unit of Infectious Diseases, Department of Medical Sciences, University of TorinoTorino, Italy

**Keywords:** HIV, cognitive impairment, HIV-associated neurocognitive disorders, neurocognitive rehabilitation, HAND

## Abstract

The primary aim of the present study was to evaluate the efficacy and stability over time of a cognitive rehabilitation protocol (restorative and compensatory approach) in HIV/AIDS patients with HIV-associated Neurocognitive Disorder (HAND). At baseline, 32 HIV/AIDS patients (16 with and 16 without HAND) were assessed with a neuropsychological battery (i.e., pre-assessment) consisting of 22 tests covering eight cognitive domains. Then, the experimental group was administered over 4 months a cognitive rehabilitation protocol aimed at improving four cognitive domains by means of eight paper and pencil/computer-based exercises. The control group received guideline-adherent clinical care (i.e., standard of care). At the end of the cognitive treatment, both groups were re-administered the neuropsychological battery (i.e., post-assessment). Additionally, 6 months after post-assessment, the experimental group was given the same neuropsychological battery (i.e., follow up-assessment). In order to test the efficacy of the cognitive rehabilitation protocol, we compared between groups the results of the neuropsychological battery at the pre- and post-assessments. In order to evaluate the stability over time, the effects of the cognitive rehabilitation protocol was examined comparing within the experimental group the results of the neuropsychological battery at post- and follow up-assessments. Our results show that the two groups did not differ at the pre-assessment, but differed at post-assessment. Specifically, the experimental group showed a significant improvement in five domains (Learning and memory, Abstraction/executive functioning, Verbal fluency, Attention/working memory, and Functional), whereas the control group significantly worsened in the same domains. The improvement of the experimental group did not change in the follow up-assessment in two domains (Abstraction/executive functioning, Attention/working memory, and Functional). Overall, these findings support the efficacy and, to some extent, the stability over time of our cognitive rehabilitation protocol.

## Introduction

Combination antiretroviral therapy has extended the survival of patients living with Human Immunodeficiency Virus (HIV) and, as a result, HIV is becoming a chronic disease. At the same time, however, patients appear to be more commonly affected by physical, social and cognitive disabilities (e.g., O'Brien et al., 2014) than the general population. For instance, in a population-based sample of people living with HIV, over 80% of patients reported at least one impairment, activity limitation or social participation restriction (Rusch et al., 2004). One of the most common causes of disability is HIV-associated Neurocognitive Disorder (HAND; Heaton et al., 2010). The largest HAND study to date reported deficits in 52% of HIV-seropositive adults (Heaton et al., 2010). Since, these disabilities can affect clinical outcomes, cognitive rehabilitation may be a key step in the maintenance or improvement of quality of life of HIV patients.

Consensus research criteria for classifying HAND were published in 2007 (Antinori et al., 2007). Specifically, the authors proposed three categories: Asymptomatic Neurocognitive Impairment (ANI), Mild Neurocognitive Disorder (MND), and HIV-Associated Dementia (HAD). The taxonomy is based on objective diagnostic criteria and account for daily functioning. The diagnosis of mild (MND) or severe (HAD) symptomatic HAND, for instance, requires a functional decline in at least two cognitive domains.

While the incidence of HAD has been reduced with combination antiretroviral therapy, the prevalence of less severe forms of neurocognitive disorders (i.e., ANI and MND) has remained relatively stable. Further, a large longitudinal study found that both ANI and MND were associated with cognitive worsening (Grant et al., 2014). One explanation for the persistence of mild HAND is antiretroviral neurotoxicity (Giunta et al., 2011; Robertson et al., 2012). Such toxicity may occur via direct drug effects on neurons and glia or indirectly via drug-linked effects on other organs, such as the cardiovascular system (Underwood et al., 2015).

The development of treatments for HAND is an important unmet clinical need. With regard to cognitive rehabilitation, three studies have been published to date (Boivin et al., 2010; Becker et al., 2012; Vance et al., 2012) but none employed HAND diagnoses as inclusion criteria and all used the so-called restorative approach that aims to restore the neural circuits underlying impaired cognitive processes by means of practice and focused training exercises. These studies reported positive effects on visual learning (Boivin et al., 2010) and speed of information processing (Boivin et al., 2010; Vance et al., 2012). However, the improvement did not extend to other ecologically relevant cognitive domains, such as executive functioning or working memory, and the durability of the benefits was not strongly addressed.

In the present study, we aimed to examine the efficacy and durability of a cognitive rehabilitation treatment for HAND in HIV+ adults taking suppressive antiretroviral therapy. To maximize the ecological impact of the training, our intervention combined the restorative approach of prior studies with the compensatory approach known to better enable learning of new strategies and minimizing the impact of remaining impairment (Robertson and Murre, 1999; Cicerone et al., 2005; Dams-O'Connor et al., 2009). We predicted a significant improvement of the neuropsychological picture in HAND-treated, respect to HAND-untreated patients. Additionally, we predicted the stability over time of these effects.

## Materials and methods

### Participants

Thirty-two patients (16 with and 16 without HAND) in care to the Infectious Disease Unit “Division A,” Amedeo of Savoia Hospital (Turin, Italy) provided written informed consent to participate in the study, which was approved by the Local Bioethics Committee (ASL TO2). The study did not include any pharmacological intervention, and it was performed in accordance with the ethical standards described in the Declaration of Helsinki (World Medical Association, 2013).

Patients were randomized 1:1 to either the experimental group or the control group. The two groups did not differ in demographic, clinical, or treatment characteristics (all *p*>0.05, see Table 1).

**Table 1:** **Demographical/Clinical data of the two groups of patients and their statistical comparisons at baseline (pre-assessment)**.

	**Experimental group**	**Control group**	***p***
Sample size	16	16	>0.05
Plasma HIV-RNA^b^ (≤ 50 c/mL)^2^	16	16	>0.05
Years HIV infected^a^	11.25 (5.8)	8.75 (6)	>0.05
Current CD4+ T-cell count^c^	539 (299–611)	614 (369–810)	>0.05
Nadir CD4 T-cell count^c^	212 (100–273)	177 (57–265)	>0.05
HCV seropositive^b^ (%)	3 (19)	1 (6)	>0.05
Number of current antiretrovirals^c^	3 (3–4)	3 (3–4)	>0.05
CPE (CNS penetration effectiveness score) of current regimen^a^	7.1 (2)	7.5 (1.3)	>0.05
Age (years)^c^	47.5 (12.2)	50 (8.4)	>0.05
Ethnicity (Cau)^b^	16 (100)	14 (87.5)	>0.05
Education^c^	10 (3)	9 (3.9)	>0.05
Gender (Women)^b^	5 (31)	3 (19)	>0.05

*Mean (SD) Median (IQR) or Number (%)*.

a *Mean (Standard deviation)*.

b *Number (%)*.

c *Median (Inter-quartile range)*.

Both groups were administered a pre-assessment neuropsychological battery (see below). Then, the experimental group was given the cognitive rehabilitation intervention for 4 months (see below for the full description) and the control group was given standard of care. At the end of the cognitive treatment, both groups were re-administered (post-assessment) the neuropsychological battery. The experimental group was also given the neuropsychological battery 6 months after the post-assessment (follow up) in a within-subjects randomized order. Raw scores on each test are reported in Table 2. Scores were corrected for age, educational level and gender using Italian normative data. Since the Grooved Pegboard Test has no standardization, it is not reported.

**Table 2:** **Groups' pre-, post-, and follow up- raw/T-scores on each test/domain of the neuropsychological battery**.

**Time**	**Domain/Test**	**Experimental group**		**Control group**
		**Raw scores**	**T scores**	**Raw scores**
Pre-assessment	*Screening*		50.2 (5.3)	
	3Q	1.1 (1.1)	48.1 (11.1)	0.7 (0.9)
	MMSE	27.6 (1.27)	5.3 (8.3)	27.5 (1.4)
	IHDS	9.3 (1.7)	51.2 (10.5)	8.9 (1.6)
	*Speed Information Processing*		49.8 (7.9)	
	TMT-A	45.9 (20.5)	49.8 (10.1)	44.9 (20.6)
	STROOP-T	28.8 (11.4)	49.8 (11.1)	28.5 (9.4)
	Learning & Memory		51.1 (7.7)	
	RAVLT-IR	39.7 (9.3)	51.6 (9.4)	36.5 (10.1)
	RAVLT-DR	8.3 (2.8)	51.3 (8.9)	7.4 (3.5)
	ROCF-DR	10.4 (5.2)	50.3 (11.1)	10.1 (4.3)
	*Abstraction/Executive Functioning*		50.4 (5.4)	
	TOL	16.9 (1.5)	51.1 (10.2)	16.6 (1.5)
	STROOP-E	2.75 (1.8)	50.8 (7.5)	3.2 (3)
	TMT-B	149 (53.4)	52.9 (7.3)	190.7 (84.8)
	FAB	13.3 (3.3)	49.9 (12.4)	13.3 (1.98)
	ROCF-C	26.9 (5.5)	47.3 (10.6)	29.7 (4.6)
	*Verbal Fluency*		51.9 (8.2)	
	FAS	28.6 (9.3)	53 (10.8)	23.3 (7.3)
	VS	3.2 (0.6)	50.9 (10.5)	3.1 (0.5)
	*Attention/Working Memory*		50.6 (5.3)	
	CORSI	3.6 (0.8)	47.8 (9.98)	3.9 (0.75)
	DS	6.6 (1.9)	50.8 (9.6)	6.3 (2)
	TMT-BA	103.1 (41.2)	53.2 (6.4)	144.4 (77.4)
	*Functional*		49.6 (9.6)	
	IADL	7.2 (0.8)	49.6 (9.6)	7.2 (0.9)
	*Mental Health*		47.7 (10.3)	
	PHQ-9	10.4 (6.8)	47.9 (10.5)	7.6 (6)
	GAD-7	9.6 (6.2)	47.6 (10.9)	6.9 (4.9)
Post-assessment	*Screening*		53.3 (4.2)	
	MMSE	28.2 (0.8)	52.6 (6.6)	27.5 (1.6)
	IHDS	10 (0.7)	54 (4.6)	9.5 (1.8)
	*Speed Information Processing*		51.3 (8.1)	
	TMT-A	46.4 (15)	50.8 (9.5)	48.9 (16.9)
	STROOP-T	25.7 (10.9)	51.9 (9.97)	29.8 (10.9)
	*Learning and Memory*		55.7 (6.2)	
	RAVLT-IR	44.5 (8.1)	55.4 (7.9)	33.4 (9.3)
	RAVLT-DR	9.6 (3.5)	53.8 (9.8)	6.9 (3.2)
	ROCF-DR	20.5 (4.5)	58.1 (6.8)	9.7 (3.1)
	*Abstraction/Executive Functioning*		54.5 (2.8)	
	TOL	18 (0.9)	54.3 (5.9)	16.7 (1.7)
	STROOP-E	1.6 (1.4)	54 (5.1)	3.8 (3.3)
	TMT-B	128.6 (34.1)	52.2 (5.5)	155.9 (80.3)
	FAB	15.8 (1.1)	56.5 (5.3)	13.1 (1.9)
	ROCF-C	32.4 (1.7)	55.5 (5.2)	28.7 (3.6)
	*Verbal Fluency*		56.7 (4.3)	
	FAS	37.5 (7.3)	56.8 (6.9)	23 (8.3)
	VS	4 (0.5)	56.5 (6.8)	3.1 (0.6)
	*Attention/Working Memory*		54.4 (4.9)	
	CORSI	4.7 (0.8)	53.4 (9.8)	4.1 (0.8)
	DS	9 (1.5)	56.4 (6.3)	6.1 (2)
	TMT-BA	82.2 (28.3)	53.4 (5.2)	119.1 (66.9)
	*Functional*		53.9 (5.6)	
	IADL	7.8 (0.4)	53.9 (5.6)	7.2 (0.9)
	*Mental Health*		49.5 (11.1)	
	PHQ-9	8.7 (6.3)	49.5 (10.9)	8.2 (5.4)
	GAD-7	6.8 (5.8)	49.6 (11.7)	6.4 (4.2)
Follow up-assessment	*Screening*		50 (5)	
	MMSE	28.8 (0.7)	50 (10)	
	IHDS	10.8 (0.9)	50 (10)	
	*Speed Information Processing*		50 (7.9)	
	TMT-A	38.2 (15.2)	50 (10)	
	STROOP-T	25.4 (8.5)	50 (10)	
	Learning and Memory		50 (7.9)	
	RAVLT-IR	49.8 (13.6)	50 (10)	
	RAVLT-DR	10.4 (2.6)	50 (10)	
	ROCF-DR	21.5 (5.1)	50 (10)	
	*Abstraction/Executive Functioning*		50 (3.6)	
	TOL	18.2 (0.7)	50 (10)	
	STROOP-E	1.9 (1.1)	50 (10)	
	TMT-B	114.4 (24.2)	50 (10)	
	FAB	16.1 (1)	50 (10)	
	ROCF-C	31.9 (2)	50 (10)	
	*Verbal Fluency*		50 (8.5)	
	FAS	38.2 (11.4)	50 (10)	
	VS	3.5 (0.6)	50 (10)	
	*Attention/Working Memory*		50 (6.9)	
	CORSI	4.7 (0.5)	50 (10)	
	DS	8.7 (1.2)	50 (10)	
	TMT-BA	73.8 (19.3)	50 (10)	
	*Functional*		50 (10)	
	IADL	7.9 (0.25)	50 (10)	
	*Mental Health*		50 (9.5)	
	PHQ-9	8.5 (4.8)	50 (10)	
	GAD-7	6.7 (3.6)	50 (10)	

*Scores are corrected for age, educational level and gender in the Italian population*.

*3Q, Simioni's 3 question test, < 1 pathological; MMSE, Mini Mental State Examination, < 26 pathological; IHDS, International HIV Dementia Scale, < 10 pathological; TMT-A, Trial Making Test Part A, >68 pathological; STROOP-T, Stroop Time, ≥31.66 pathological; RAVLT-IR, Rey Auditory Verbal Learning Test Immediate Recall, < 32.26 pathological; RAVLT-DR, Rey Auditory Verbal Learning Test Delayed Recall, < 5.8 pathological; ROCF-DR, Rey-Osterrieth Complex Figure Delayed Recall, ≤ 11.22 pathological; TOL, Tower of London, < 15 pathological; STROOP-E, Stroop Errors, ≥2.81; TMT-B, Trial Making Test Part B, >177 pathological; FAB, Frontal Assessment Battery, < 14.4 pathological; ROCF-C, Rey-Osterrieth Complex Figure Copy, ≤ 30.04 pathological; FAS, Phonemic Fluency; < 21.33 pathological; VS, Verbal Span, < 3.5 pathological; CORSI, Corsi block-tapping test, < 3.76 pathological; DS, Digit Span, < 8 pathological; TMT-BA, Trial Making Test Part BA, > 111 pathological; IADL, Instrumental Activity of Daily Living Questionnaire, ≤ 6 pathological; PHQ-9, Patient Health Questionnaire, >15 pathological; GAD-7, Generalized Anxiety Disorder, >15 pathological*.

*Mean (Standard error)*.

### Neuropsychological battery

Initially, 220 consecutive HIV/AIDS patients were administered three different *screening tests* of the neuropsychological battery in a between-subjects randomized order:

- Mini Mental State Examination (MMSE; Folstein et al., 1975)
- International HIV Dementia Scale (IHDS; Sacktor et al., 2005)
- Simioni's 3 question test (3Q; Simioni et al., 2010)

Then, patients with a score below the cut off in MMSE, and/or at three questions test, and/or at the IHDS were administered in a between-subjects randomized order the rest of the neuropsychological battery (*N* = 110) composed by 19 tests covering seven different cognitive domains:

- *Speed of information processing*
  ◦ Trail Making Test Part A (TMT-A, Giovagnoli et al., 1996)
  ◦ Stroop Color Test-Time (STROOP-T, Caffarra et al., 2002a)
- *Learning and Memory*
  ◦ Rey Auditory Verbal Learning Test Immediate Recall (RAVLT-IR, Carlesimo et al., 1996)
  ◦ Rey Auditory Verbal Learning Test Delayed Recall (RAVLT-IR, Carlesimo et al., 1996)
  ◦ Rey-Osterrieth Complex Figure Delayed Recall (ROCF-DR, Caffarra et al., 2002b)
- *Abstraction/Executive Functioning*
  ◦ Tower of London simplified version (ToL; Allamanno et al., 1987)
  ◦ Stroop Color Test-Errors (STROOP-E, Caffarra et al., 2002a)
  ◦ Trail Making Test Part B (TMT-B, Giovagnoli et al., 1996)
  ◦ Frontal Assessment Battery (FAB, Appollonio et al., 2005)
  ◦ Rey-Osterrieth complex Figure Copy (ROCF-C, Caffarra et al., 2002b)
- *Verbal Fluency*
  ◦ Phonemic Fluency (FAS, Carlesimo et al., 1995)
  ◦ Verbal Span (VS, Spinnler and Tognoni, 1987)
- *Attention/Working Memory*
  ◦ Corsi's block-tapping Test (CORSI, Orsini et al., 1987)
  ◦ Digit Span (DS, Orsini and Laicardi, 1997)
  ◦ Trail Making Test Part BA (TMT-BA, Giovagnoli et al., 1996)
- *Motor*
  ◦ Grooved Pegboard Test Dominant and non- dominant hands (Heaton et al., 1991)
- *Functional*
  ◦ Instrumental Activity of Daily Living Questionnaire (IADL, Lawton and Brody, 1969). This latter test was administered to investigate 10 areas of autonomy in activities of daily living: ability to use the phone, grocery shopping, preparing meals, take care of the house, laundry, moving away from home, assumption drugs, use of money, work ability, work efficiency.
- *Mental Health*
  ◦ Patient Health Questionnaire (PHQ-9, Kroenke and Spitzer, 2002)
  ◦ Generalized Anxiety Disorder (GAD-7, Kroenke and Spitzer, 2002)

Sixty of 110 tested patients (54%) were diagnosed with HAND (48 ANI, 10 MND, and 2 HAD). Participants (*N* = 32) were selected among those with HAND based on specific eligibility criteria: receiving antiretroviral therapy for at least 6 months, plasma HIV RNA below 50 copies/mL for at least 6 months, CD4+ T lymphocyte count above 350 cells/μL from at least 6 months, fluent Italian speaker, and absence of severe comorbidities, including neurodegenerative or psychiatric disease, metabolic encephalopathy, psychoactive drug use, alcohol use, or head trauma.

### Cognitive rehabilitation treatment

The cognitive rehabilitation protocol included both paper-and-pencil and computer-based exercises and it was composed of eight different exercises repeated in 36 sessions (around 50 min each) over 4 months. The eight tests were administered in a randomized-within subjects order. The protocol aimed at improving with the different group of tests four cognitive domains:

- *Attention*
  ◦ Time Pressure Management (Fasotti et al., 2000)
    ▪ Five minute paper-and-pencil exercise to improve functional impairments related to slowed information processing and complex attention. Patients learn compensatory strategies as, for instance, allowing sufficient time to manage a task
  ◦ Attention Process Training Task (Cicerone et al., 2005)
    ▪ Five minute paper-and-pencil exercise organized hierarchically to improve different components of attention: sustained, selective, alternating and divided attention. Patients learn compensatory strategies as, for instance, removing environmental distractors or employing using cues to maintain attention
- *Visual-verbal memory and learning*
  ◦ COG.I.T.O. (open platform. ASPHI and San Camillo Hospital, Turin
    ▪ Ten minute computer-based exercise to improve attention/visual-spatial memory. Patients have to encode daily-use objects in domestic environments (i.e., kitchen, bedroom, garage). Then, objects disappear and they have to put them back in their correct location, or recognizing them among distractors and relocating them, or writing their names and relocating them. From session to session, the available time decreases whereas the number of objects increases.
  ◦ Errorless Learning (Ehlhardt et al., 2005)
    ▪ Five minute paper-and-pencil exercise providing sufficient cues during training so that patients can only give correct responses. Then, cues are progressively sequentially reduced
  ◦ Process-Oriented Memory Learning (Huildebrandt et al., 2006)
    ▪ Five minute paper-and-pencil exercise to improve strategies adapted to different situations with memory requirements (e.g., practice, managing interferences between acquisition and recall, principles to optimize memory performance)
- *Executive Functioning and Working Memory*
  ◦ Metacognitive Strategy Training (Kennedy et al., 2008)
    ▪ Five minute paper-and-pencil exercise to improve daily problem-solving abilities (e.g., use of metacognitive approaches incorporating emotional self-regulation strategies which facilitates clear thinking)
  ◦ Goal Management Training (Levine et al., 2000)
    ▪ Five minute paper-and-pencil exercise to improve the ability to stop and think about what one is doing, identifying a specific goal, delineating the steps or achieve a goal and evaluating the outcomes
- *Metacognitive Awareness*
  ◦ Increased Awareness (10 min)
    ▪ One minute at the beginning of each session, 1 min at the end of each exercise for 10 min to improve awareness of neurocognitive deficits (Dams-O'Connor and Gordon, 2010).

## Results

Raw corrected scores on each neuropsychological test at pre pre-, post-, and follow up- were standardized (T-score). When necessary, the standard scores were reversed in order to keep the interpretation of all tests in the same direction, namely higher scores reflecting a better performance. Then the standard scores were averaged within each domain.

In order to evaluate the efficacy of the cognitive rehabilitation protocol, we performed a repeated measures ANOVA on each domain of the neuropsychological battery with the mean standard scores as dependent variables, TIME (two levels: pre-assessment, post-assessment) as within-subjects factor, and GROUP (two levels: experimental, control) as between-subjects factor.

**Figure 1:**
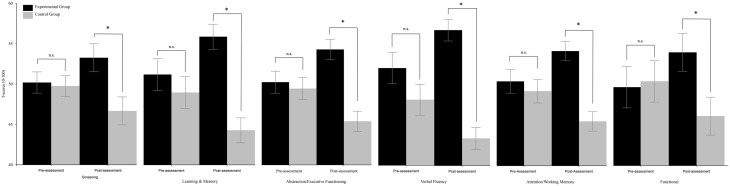
**Between-groups comparisons (domains) along time (pre- vs. post-assessment)**. Error bars represent standard errors. Asterisks indicate significant comparisons, n.s. indicate non-significant comparisons.

Respect to “Screening,” the TIME x group interaction resulted to be significant [*F*_(1, 30)_ = 7.05, *p* = 0.013]. *Post-hoc* comparisons (Duncan) revealed that the two groups did not differ at pre-assessment (experimental group: mean = 50.23, *SE* = 1.32; control group: mean = 49.77, *SE* = 1.31), but differed (*p* = 0.007) at post-assessment (experimental group: mean = 53.3, *SE* = 1.74; control group: mean = 46.7, *SE* = 1.74); see Figure 1. The analysis on “Speed information processing” was not significant. As regards “Learning and memory,” the TIME × GROUP interaction was significant [*F*_(1, 30)_ = 31.58, *p* < 0.0001]. *Post-hoc* comparisons (Duncan) revealed that the two groups did not differ at pre-assessment (experimental group: mean = 51.11, *SE* = 1.96; control group: mean = 48.89, *SE* = 1.95), but differed (*p* = 0.0002) at post-assessment (experimental group: mean = 55.75, *SE* = 1.54; control group: mean = 44.24, *SE* = 1.54) because the mean score of the experimental group significantly (*p* = 0.0005) increased and the mean score of the control group significantly (*p* = 0.0001) decreased (see Figure 1). The TIME × GROUP interaction was significant [*F*_(1, 30)_ = 21.42, *p* < 0.0001 also for “Abstraction/Executive Functioning.” Duncan *post-hoc* showed that the groups did not differ at pre-assessment (experimental group: mean = 50.41, *SE* = 1.9; control group: mean = 49.6, *SE* = 1.38), but differed (*p* < 0.001) at post-assessment (experimental group: mean = 54.51, *SE* = 1.27; control group: mean = 47.48, *SE* = 1.27) because the mean score of the experimental group significantly (*p* = 0.003) increased, and the mean score of the control group significantly (*p* = 0.003) decreased (see Figure 1). The same was true for the “verbal fluency” in which the TIME × GROUP interaction was significant [*F*_(1, 30)_ = 11.45, *p* = 0.002]. Duncan *post-hoc* analysis revealed that groups did not differ at pre-assessment (experimental group: mean = 51.95, *SE* = 1.91; control group: mean = 48.05, *SE* = 1.91), but differed (*p* < 0.0001) at post-assessment (experimental group: mean = 56.66, *SE* = 1.34; control group: mean = 43.34, *SE* = 1.34) because the mean score of the experimental group significantly (*p* = 0.02) increased and the mean score of the control group significantly (*p* = 0.02) decreased (see Figure 1). The TIME × GROUP interaction was significant [*F*_(1, 30)_ = 13.8, *p* = 0.001] also for “Attention/Working memory.” Duncan *post-hoc* analysis revealed that groups did not differ at pre-assessment (experimental group: mean = 50.62, *SE* = 1.49; control group: mean = 49.38, *SE* = 1.49), but differed (*p* = 0.0001) at post-assessment (experimental group: mean = 54.42, *SE* = 1.22; control group: mean = 45.58, *SE* = 1.22) because the mean score of the experimental group significantly (*p* = 0.016) increased and the mean score of the control group significantly (*p* = 0.016) decreased. The same was true for “Functional” in which the TIME × GROUP interaction was significant [*F*_(1, 30)_ = 12.91, *p* = 0.001]. Duncan *post-hoc* revealed that groups did not differ at pre-assessment (experimental group: mean = 49.64, *SE* = 2.54; control group: mean = 50.36, *SE* = 2.53), but differed (*p* = 0.042) at post-assessment (experimental group: mean = 53.92, *SE* = 2.31; control group: mean = 46.08, *SE* = 2.33) because the mean score of the experimental group significantly (*p* = 0.021) increased and the mean score of the control group significantly (*p* = 0.021) decreased. The analysis on “Mental Health” was not significant.

**Figure 2:**
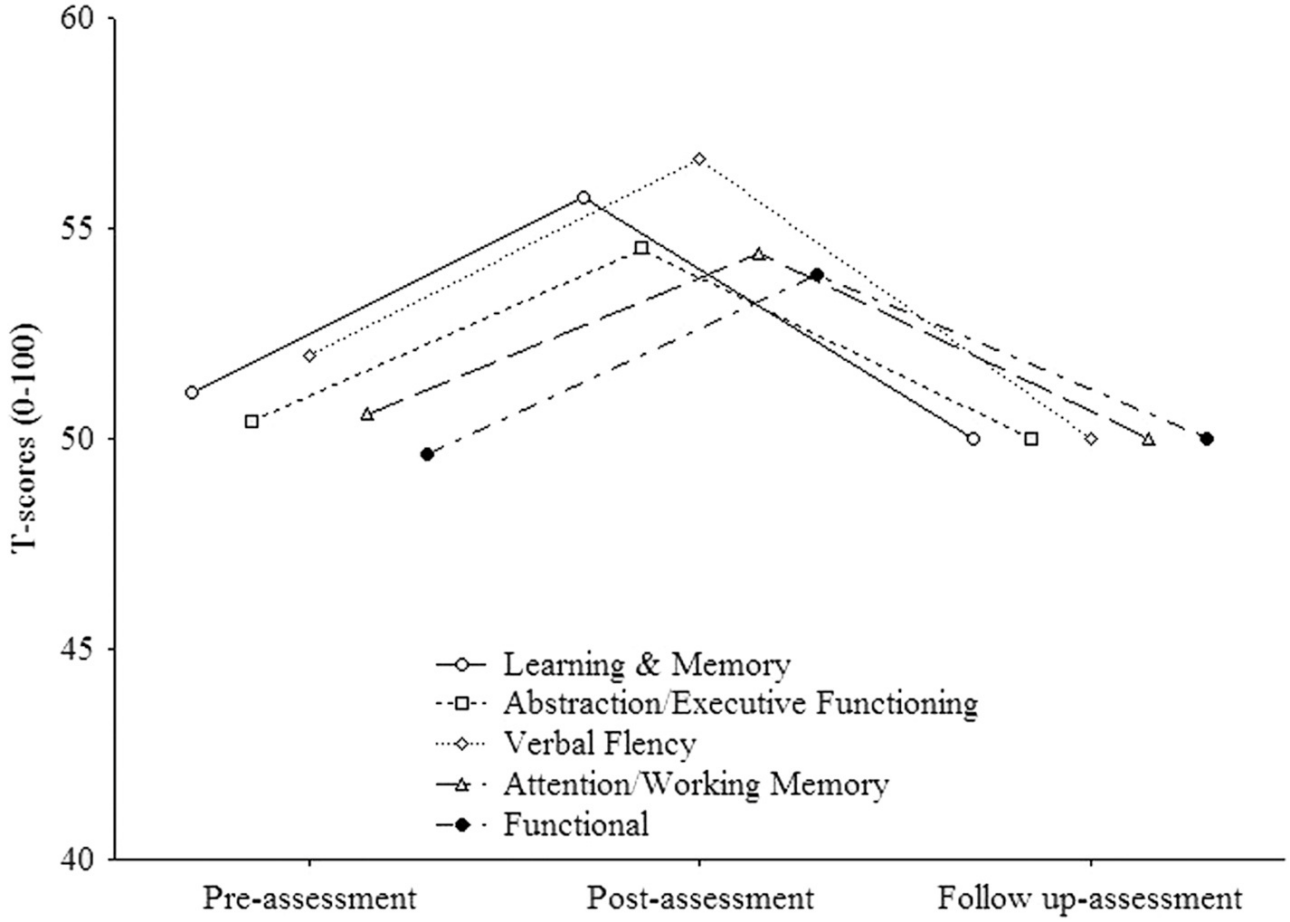
**Experimental group trends (improved domains) along time (post- vs. follow up-assessment**.

In order to evaluate whether the treatment was able to induce permanent effects, we performed in the experimental group a within subject ANOVA on each domain of the neuropsychological battery which improved from pre- to post assessments. The mean standard scores were employed as dependent variables, and TIME (three levels: pre-assessment, post-assessment, follow up-assessment) as within-subjects factor. The analysis on “Learning and Memory” (see Figure 2) resulted to be significant [*F*_(2, 30)_ = 8.92, *p* < 0.001]. *Post-hoc* comparisons (Duncan) revealed that the mean score significantly (*p* = 0.003) improved from pre- (mean = 51.1, *SE* = 1.94) to post- (mean = 55.75, *SE* = 1.55) assessment and worsened (*p* < 0.001) from post- to follow up-assessment (mean = 50, *SE* = 2.1). Also the analysis on “Abstraction/executive functioning” (see Figure 2) was significant [*F*_(2, 30)_ = 5, *p* = 0.008] with the mean score significantly (*p* = 0.006) improved (Duncan) from pre- (mean = 50.4, *SE* = 1.44) to post- (mean = 54.52, *SE* = 0.7) assessment and worsened (*p* = 0.004) from post- to follow up-assessment (mean = 50, *SE* = 0.9). The same was true (see Figure 2) for “Verbal Fluency” [*F*_(2, 30)_ = 5.5, *p* = 0.01) with the mean score significantly (*p* = 0.03) improved (Duncan) from pre- (mean = 51.95, *SE* = 2.06) to post- (mean = 56.67, *SE* = 1.07) assessment and worsened (*p* = 0.005) from post- to follow up-assessment (mean = 50, *SE* = 2.1). As regards “Attention/working memory” (see Figure 2), the analysis resulted to be significant. However, *post-hoc* comparisons (Duncan) revealed that the mean score significantly (*p* = 0.03) improved from pre- (mean = 50.62, *SE* = 1.31) to post- (mean = 54.44, *SE* = 1.22) assessment but did not change (*p*>0.05) from post- to follow up-assessment (mean = 50, *SE* = 1.74). The analysis on “Functional” (see Figure 2) was not significant (*p*>0.05).

## Discussion

In the present study, we examined the efficacy of a cognitive rehabilitation protocol that included both restorative and compensatory approaches for treatment of HAND. Our results show discordant clinical evolution in five out of eight domains in participants who received the intervention compared with those who did not: treated patients improved, untreated patients worsened. Additionally, the improvement proved to be stable over time in four of five domains.

Within the context of HIV, rehabilitation is a dynamic set of activities that can benefit the disease as well as linked social limitations and dysfunctions (Worthington et al., 2005). With the graying of the HIV population, the demand for rehabilitation services will likely increase in the near future (O'Brien et al., 2014). Since a substantial number of HIV patients have cognitive impairment (e.g., Heaton et al., 2010), expansion in rehabilitation services should include cognitive rehabilitation.

One definition of cognitive rehabilitation is a systematic, functionally oriented set of therapeutic activities. Cognitive improvement is achieved by both strengthening previously learned behavioral patterns and establishing new, compensatory ones (Bergquist and Malec, 1997). This is possible because of the brain plasticity, the changes of brain organization that subserve short- and long-term behavioral modifiability. These changes can be structural (i.e., remodeling of the brain's physical structures) or physiological (i.e., dynamic adjustment of cellular processes such as synapse formation that modulate conductance or resistance to impulse transmission; see, for instance, Berlucchi, 2011). These changes can include adaptation to novel environments, maturation, different learning types, and compensatory changes in response to functional loss (see, for instance, Berlucchi, 2011).

Successful cognitive rehabilitation treatment typically targets different cognitive abilities, such as attention, memory, perception, learning, and executive functioning. Metacognitive awareness, emotional regulation, social skills and community integration are other, important targets. The main aim of cognitive rehabilitation is to significantly reduce the impact of disease on daily living. More specific aims depend on the nature and severity of the individual cognitive, behavioral, physical, and emotional difficulties as well as on the specific individual premorbid achievements (Dams-O'Connor and Gordon, 2010).

With HAND, HIV mainly affects fronto-striatal-thalamocortical circuitry, often being associated with decreased white matter volume (e.g., Thompson et al., 2005). As a consequence, cognitive deficits can cover a wide range of abilities. For instance, patients may develop impairments of visual/verbal working memory (Martin et al., 2001) as a consequence of central executive dysfunctions (Hinkin et al., 2002). Additionally, patients may show working memory impairments that can influence even higher-level cognitive abilities and daily activities (Martin et al., 2004). Nonetheless, HIV is frequently associated with difficulties of attentional processes (Hinkin et al., 2002), which, in turn, may negatively affect adherence to antiretroviral therapy (Levine et al., 2005, 2008). As a result, patients can experience multiple personal and professional difficulties and are frequently unemployed (Heaton et al., 1994; van Gorp et al., 2007).

In the present study, we aimed to maximize the effects of a cognitive rehabilitation intervention by training patients in both restorative (i.e., COG.I.T.O) and compensatory abilities (Time Pressure Management, Attention Process Training Task, Errorless Learning, Process-Oriented Memory Learning, Metacognitive Strategy Training, Goal Management Training, Increased Awareness). Each exercise was organized and administered in different steps with progressively increasing difficulty, suitable for gradual improvement of performance and for learning of any targeted ability. As written above, we found a significant improvement in the experimental, respect to the control, from pre- to post-assessments in Learning and memory, Abstraction/executive functioning, Verbal fluency, Attention/working memory, and Daily functioning. The improvement in Attention/working memory indirectly suggests benefits for daily functioning (working memory) and medication adherence (attention). The improvement in Learning and memory could explain the improvements in self-reported IADLs at post-assessment as well as the better work efficiency reported by treated patients. In contrast, the control group significantly worsened in the same domains.

At follow up-assessment, the cognitive improvement were stable (i.e., no significant difference respect to post-assessment) in two domains. It is worth noticing that previous long-term (i.e., 5 months to 1 year) benefits of cognitive rehabilitation (Tesar et al., 2005; Svendsen and Teasdale, 2006; Fink et al., 2010; Stuifbergen et al., 2012), have been related to patients continued use of learned strategies in ecological situations, as well as to the relevance of the intervention to the patient's daily functioning (Cicerone et al., 2000). Then, patients of experimental group used learned strategies in ecological contexts of daily living. However, in the other three domains, the experimental group's performance significantly worsened (and, indeed, this is also the overall trend). Broadly speaking, this is not surprising but, rather, consistent with the fact that most long-term follow up studies on the effects of acquired brain injuries show also some persisting effects (Klonoff et al., 1993; Dikmen et al., 2003; Wood and Rutterford, 2006). Additionally, ARV neurotoxicity and the potential HIV activities within the central nervous system acts against the cognitive rehabilitation process. Indeed, this might also explain, at least in part, the above-mentioned fact that the control group worsened from pre- to post-assessment.

Our study had several limitations. First, the sample size is relatively small. Hence, further studies with larger samples are required to validate the findings. Secondly, and more importantly, the control group was administered standard of care only but not a structured activity, raising the possibility that repeating *per se* the cognitive rehabilitation protocol might have a role in the improvement observed in the experimental group. This possibility is countered by the fact that benefit was not observed in all domains, which would have been seen if the improvement was solely due to practice. Still, this remains a limitation of our investigation and future studies should employ active control activities such as generalized compensatory cognitive training or low cognitive demand computer activities (Weber et al., 2013). Interestingly, those studies might address the effects of different kind of interventions: restorative approaches vs. compensatory interventions, purely computer-based vs. purely paper-based. Beyond the presence of a control condition, such strategies would better enable proof of concept for the use of cognitive rehabilitation in HAND.

In conclusion, our intervention was associated with better cognitive performance. According to a recent call to action (Weber et al., 2013), future studies should prioritize development of specific cognitive rehabilitation interventions for HAND, particularly with emphasis on two issues. First, while our center is similar to other Italian centers, a specific battery of standardized tests for HIV populations should be developed in Italy to generalize our findings to different countries. Secondly, the impact of cognitive rehabilitation on daily living, quality of life, and medication adherence has to be clarified. Since unawareness is in general a significant barrier to treatment (Ownsworth et al., 2002) and since more than 50% of people with HIV have poor insight of their cognitive deficits (Weber et al., 2013), more specific measures and more sensitive interventions should be developed.

### Conflict of interest statement

The authors declare that the research was conducted in the absence of any commercial or financial relationships that could be construed as a potential conflict of interest.
